# Impure Glycerine

**Published:** 1868-06

**Authors:** 


IMPURE GLYCERINE.—Mr. Henry Bower, of Philadelphia, writes to the *Chemical News* on this topic as follows:

"The writer of the article on Glycerine in *Kunst und Gewerbeblatt* is correct in attributing the acrid, irritating properties of some glycerine to the mode of preparation; but I have seen distilled glycerine which was quite as unsuitable for medicinal or surgical purposes as any spoken of. The volatile fatty acids, and ethers, which exist in crude glycerine, are sometimes condensed with the glycerine, and these have very irritating properties.

In the glycerine which is made without distillation, the volatile acids and ethers exist, but not in the same state as after distillation, the heat required for this process decomposing them into some modification of their original state.

The great cause of irritation in glycerine which has not been properly prepared, is the presence of oxalic acid and of formic acid; these are produced by the action of sulphuric acid upon the glycerine, forming the first mentioned acid, and this in its turn acts upon the glycerine, giving rise to formic acid, the irritating properties of which are well known.

The nitrate of silver test I have always considered most reliable. Glycerine which shows no reaction with this salt may be considered suitable for all uses, as it indicates not only the presence of chlorine or chlorides, but is, as well, reduced by acids, which may exist in the glycerine."

